# Allelic diversity and phylogeny of *homB*, a novel co-virulence marker of *Helicobacter pylori*

**DOI:** 10.1186/1471-2180-9-248

**Published:** 2009-12-02

**Authors:** Mónica Oleastro, Rita Cordeiro, Armelle Ménard, Yoshio Yamaoka, Dulciene Queiroz, Francis Mégraud, Lurdes Monteiro

**Affiliations:** 1 Departamento de Doenças Infecciosas, Instituto Nacional Saúde Dr Ricardo Jorge, Av. Padre Cruz, 1649-016 Lisboa, Portugal; 2 INSERM U853, 33076 Bordeaux, France; 3 Université Victor Segalen Bordeaux 2, Laboratoire de Bactériologie, Bat. 2B RDC Zone Nord, 33076 Bordeaux cedex, France; 4 Department of Medicine, Michael E. DeBakey Veterans Affairs Medical Center and Baylor College of Medicine, 2002 Holcombe Blvd. Houston, Texas 77030, USA; 5 Laboratório de Pesquisa Bacteriologia, Faculdade de Medicina, UFMG, Av. Alfredo balena, 190 S/4026 30130-100, Belo Horizonte, Brazil

## Abstract

**Background:**

The *homB *gene is a *Helicobacter pylori *disease-marker candidate, strongly associated with peptic ulcer disease, while *homA*, its paralogue gene with 90% sequence identity, is correlated with non-ulcer dyspepsia. The HomB encoded outer membrane protein was shown to contribute to the proinflammatory properties of *H. pylori *and also to be involved in bacterial adherence.

This study investigated the distribution of *homB *and *homA *genes in 455 *H. pylori *strains from East Asian and Western countries, and carried out sequence comparison and phylogenetic analyses.

**Results:**

Both *homB *and *homA *genes were heterogeneously distributed worldwide, with a marked difference between East Asian and Western strains.

Analysis of *homB *and *homA *sequences revealed diversity regarding the number of copies and their genomic localization, with East Asian and Western strains presenting different genotypes. Moreover, *homB *and *homA *sequence analysis suggests regulation by phase variation. It also indicates possible recombination events, leading to gene duplication or *homB*/*homA *conversion which may as well be implicated in the regulation of these genes. Phylogenetic reconstruction of *homB *and *homA *revealed clustering according to the geographic origin of strains. Allelic diversity in the middle region of the genes was observed for both *homB *and *homA*, although there was no correlation between any allele and disease. For each gene, a dominant worldwide allele was detected, suggesting that *hom*B/*hom*A allelic variants were independent of the geographical origin of the strain. Moreover, all alleles were demonstrated to be expressed *in vivo*.

**Conclusion:**

Overall, these results suggest that *homB *and *homA *genes are good candidates to be part of the pool of *H. pylori *OMPs implicated in host-bacteria interface and also contributing to the generation of antigenic variability, and thus involved in *H. pylori *persistence.

## Background

*H. pylori *infection is implicated in the development of several gastroduodenal diseases, ranging from chronic active gastritis and dyspepsia to peptic ulcer disease (PUD), and associated with an increased risk for gastric cancer [1]. The virulence of the infecting strain influences the severity of the clinical outcome, and disease associations have been proposed for the *cag *pathogenicity island (PAI), *vacA *and several genes encoding outer membrane proteins (OMP) [2-7]. Indeed, bacterial factors which modulate interactions with human cells, such as OMPs, have been involved in the pathophysiology of the infection caused by *H. pylori*. These proteins can contribute to the colonization and persistence of *H. pylori*, as well as influence the disease process [5-7]. PUD usually occurs after a long-term *H. pylori *infection. However, the disease can develop earlier, and rare cases have been observed in children, suggesting that the *H. pylori *strains involved are more virulent.

Recently, a novel virulence-associated OMP-coding gene, *homB*, was identified in the genome of a *H. pylori *strain isolated from a five-year old child with a duodenal ulcer [8]. The *homB *gene was associated with an increased risk of PUD as well as with the presence of other *H. pylori *disease-related genes: *cagA*, *babA*, *vacA*s1, *hopQ*I and functional *oipA *[8-10].

Several *H. pylori *strains carry a paralogue of *homB*, the *homA *gene, which presents more than 90% identity to *homB *[11]. Interestingly, *homA *was more frequently found in strains isolated from non-ulcer dyspepsia (NUD), and was associated with the less virulent *H. pylori *genotypes i.e. *cagA*-negative and *babA*-negative, *vacA*s2, *hopQ*II and a non-functional *oipA *gene [9,10].

Both *homB *and *homA *genes can be found as a single or double-copy in the *H. pylori *genome, or alternatively a copy of each gene can be present within a genome, in two conserved loci [9]. When present as a single copy, the gene always occupies the *HP0710*/*jhp0649 *locus, while when present as a double-copy, *homA *and *homB *occupy indifferently the *HP0710*/*jhp0649 *or *jhp0870 *loci [9], according to the numbering of the 26695 and J99 strains, respectively [12,13]. Furthermore, among all possible *homB *and *homA *combinations, the genotype the most significantly associated with PUD was the double-copy of *homB*, while a single copy of *homA *was the genotype the most associated with NUD [9,10].

*In vitro *studies revealed that the HomB protein is expressed as an OMP and is antigenic in humans. Moreover, HomB induces activation of interleukin-8 secretion and is involved in adherence to human gastric epithelial cells; these two phenomena being more pronounced in strains carrying the *homB *double-copy genotype [9].

Taken together, these data suggest that *homB *gene is a new co-marker for *H. pylori *virulence and that the mechanism underlying the involvement of HomB in inflammation is bacterial adherence.

The present study aimed to explore the distribution of *homB *and *homA *genes in different geographical regions. Moreover, no information on *homB *and *homA *allelic variation at the population level is available to date. Thus, to better understand the diversity and evolution of these two *H. pylori *OMP-coding genes, both comparative and phylogenetic sequence analyses were performed, using *H. pylori *strains with a different geographical background.

## Results

### Distribution of *homB *and *homA *genes in *H. pylori *strains isolated from different countries

The presence of *homB *and *homA *genes in the *H. pylori *clinical strains was determined by a single PCR with a set of primers designed on a consensus internal sequence present in both genes, which generates PCR products of 161 bp and 128 bp for *homB *and *homA*, respectively. A PCR product of one of these sizes was obtained for 449 out of 455 strains tested, suggesting that one of these genes is always present in the *H. pylori *genome. However, in six remaining cases, PCR fragments of an intermediate length were observed (146 bp for four Korean and one French strain and 152 bp for one Japanese strain), which does not relate to either the *homB *or the *homA *genotype. Although phylogenetic analysis of these PCR fragments showed that these particular sequences were closer to *homB *gene, those of the discriminating region (from 470 to 690 bp) and the entire gene (GenBank accession numbers EU910189 to EU910194) did not show a higher similarity with either *homB *or *homA*, instead the sequences were grouped by geographic origin (data not shown). These sequences were excluded from further analysis.

Analysis of the distribution of *homB *and *homA *genes in the *H. pylori *clinical strains (n = 449) from the different countries studied revealed that both genes were equally distributed among Western countries (n = 300, 56.0% for *homB *and 60.4% for *homA*). *homA *was found slightly more frequently than *homB *in strains from Portugal (n = 115, 66.5% vs 49.7%), France (n = 34, 58.9% vs 46.7%), Sweden (n = 27, 58.6% vs 41.5%), USA (n = 29, 72.4% vs 53.4%) and Brazil (n = 56, 73.4% vs 62.4%), while *homB *was more frequently found in strains from Germany (n = 20, 60% vs 45%) and Colombia (n = 19, 67.8% vs 42.8%). Among strains from East Asian countries (n = 138), *homB *was highly frequent in both Japan and Korea (n = 71, 95.9% and n = 67, 77.2%, respectively), while *homA *was more rare (5.9% and 21.2%, respectively). In strains from Burkina Faso (n = 11), both genes were highly frequent (90.9%).

### Diversity of *homB *and *homA *genes

**Figure 1:**
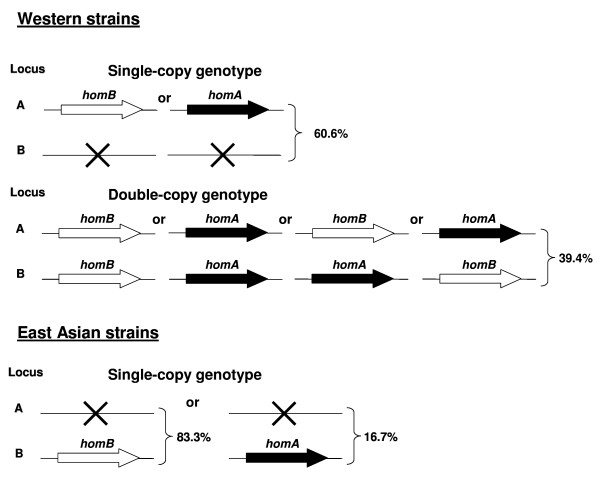
**Diversity in the number of copies and genomic localization of *homB *and *homA *in Western and East Asian *Helicobacter pylori *strains**. The percentage indicates the frequency of each type of genotype among Western and East Asian strains. **X **represents the "empty" locus.

Considering the numbering of the J99 strain, the *homA *and *homB *genes are localized at the *jhp0649 *locus (locus A) and the *jhp0870 *locus (locus B), respectively [13]. In strain 26695, only one copy of the *homA *gene is present at locus A [12], and in strain HPAG1, only one copy of the *homB *gene is present at locus A [14]. Using PCR primers located in a conserved region on the flanking genes of both A and B loci, the entire nucleotide sequence of both genes was determined for 92 clinical strains, chosen in order to represent a subgroup of each country (Portugal: 14; France: 7; Sweden, Germany, USA, and Korea: 10 each; Brazil: 11; Colombia: 9 Japan: 8; and Burkina Faso: 3) and according to their *homB*/*homA *genotype, carrying either one copy (n = 60) or two copies of *homB *and/or *homA *genes (n = 32). The analysis of 124 sequences, 71 *homB *and 53 *homA*, revealed diversity regarding the number of copies of each gene and their genomic localization between East Asian and Western strains (Fig. 1). Concerning the number of copies, strains presented either the single-copy or the double-copy genotype. The single-copy genotype was more frequently observed than the double-copy genotype in all European countries studied: Portugal (9/14 strains), France (5/7), Sweden (8/10) and Germany (8/10), as well as in Colombia (6/9), Japan (8/8) and Korea (10/10), and was independent of the clinical origin of the strains. The presence of two copies within the same strain was observed in half of the USA (5/10) isolates, and was more frequent in strains from Brazil (8/11) and Burkina Faso (3/3).

In the group of clinical strains analysed, *homB *and *homA *genes were always localized in the two loci A and B, occupying indifferently one of the loci when one copy of each gene was present within the same genome. However, in the case of a single-copy genotype, the gene was always in the same genomic position (Fig. 1): locus A in one Korean strain and in all Western strains, with the exception of three strains from US citizens of Asian origin; locus B in those three USA strains and in all Asian strains, except for the Korean strain. In the case of the single-copy genotype, the "empty" locus contained a region ranging from 236 to 573 bp with high sequence identity (88-97%) with the 3' end of both *homB *and *homA *genes.

Analysis of the entire nucleotide sequence of both *homB *and *homA *genes revealed a complete open reading frame (ORF) in 117 of the 124 sequences analyzed (94.4%). The *homB *gene size ranged from 1971 to 2013 bp and *homA *gene from 1959 to 2004 bp, leading to putative 656-670 and 652-667 residue protein lengths for HomB and HomA, respectively. With regard to the seven truncated ORFs, the four out-of-frame *homB *genes were all from NUD strains, whereas among the three out-of-frame *homA *genes, two were from NUD and one from a gastric cancer strain. These truncated ORFs were due to the presence of frameshift mutations leading to premature STOP codons, occurring in repetitive sequence motifs for three of the four *homB *sequences, which was not the case for the three out-of-frame *homA *genes. Overall, among the seven truncated cases, only one strain harboured a complete gene at the second locus, suggesting that neither HomA nor HomB are expressed *in vitro *at locus A or B for the six remaining strains.

### Phylogenetic and evolutionary analysis of *homB *and *homA *genes

**Figure 2:**
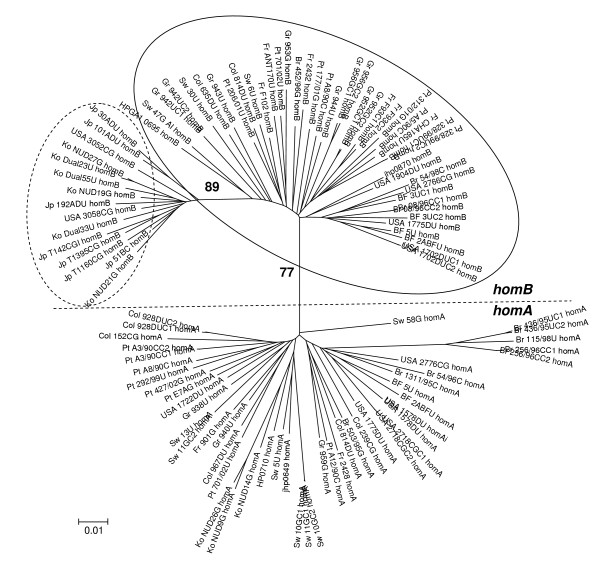
**Phylogenetic analysis of 58 *homB *and 48 *homA *sequences, obtained from *Helicobacter pylori *clinical strains from different geographical regions**. The branch length index is represented below the tree. Country of origin is located at the beginning of each strain designation (Pt, Portugal; Fr, France; Sw, Sweden; Gr, Germany; USA; Br, Brazil; Col, Colombia; Jp, Japan; Ko, Korea; BF, Burkina Faso) followed by the *homB *or *homA *status. Dotted circle, East Asian cluster; Full circle, Western cluster. The sequence of the *homB *and *homA *genes of the three *H. pylori *reference strains, 26695, J99 and HPAG1, were also included. The dotted line separates the *homB *and *homA *clusters. The numbers next to the main nodes are bootstrap values over 75% after 1000 iterations.

The phylogenetic reconstruction of *homB *and *homA *showed two independent branches for each gene (Fig. 2), suggesting a divergent evolution. Two predominant clusters corresponding to East Asian and Western countries were observed for *homB *gene pointing to a separation by geographical origin. For *homA*, the geographical segregation was not evident since this gene is rare in East Asian countries. Both *homB *and *homA *displayed a high similarity at the nucleotide level (92.8% ± 1.82 and 93.7% ± 2.20, respectively) and at the amino acid level (92.8% ± 1.82 and 94.0% ± 2.30, respectively). Furthermore, together they shared a similarity of 88.6% ± 0.006 at the nucleotide level and 89.4% ± 0.009 at the amino acid level.

The molecular distance and the nucleotide substitution rates, synonymous (Ks) and non-synonymous (Ka) substitutions, were similar for both *homB *and *homA *genes, as well as the mean Ka to mean Ks ratios (Ka/Ks) (Table 1). The type of selection operating at the amino acid level can be detected by comparing Ka and Ks [15]. Since Ka/Ks was less than 1 for both genes, the purifying selection hypothesis was tested and a significant *P *value obtained supports the hypothesis of conservation at the protein level (P_Z-Test _<0.001).

**Table 1:** Analysis of molecular distances, synonymous and non-synonymous nucleotide substitutions of *homB *(n = 67) and *homA *(n = 50), for sequences corresponding to the entire gene and to gene segments 1, 2 and 3.

	*homB *(n = 67*)				*homA *(n = 50*)			
		
	Entire gene	Segment 1	Segment 2	Segment 3	Entire gene	Segment 1	Segment 2	Segment 3
Mol. distant (nt)	0.077 ± 0.004^&^	0.067 ± 0.005	**0.124 ± 0.014**	0.075 ± 0.005	0.077 ± 0.004	0.087 ± 0.006	**0.107 ± 0.013**	0.068 ± 0.005
No. differences (nt)	138.847 ± 7.207	45.324 ± 3.377	23.737 ± 2.226	68.178 ± 4.386	136.550 ± 6.403	55.546 ± 3.750	20.104 ± 2.182	62.103 ± 4.002
Ks	0.234 ± 0.014	0.223 ± 0.024	0.234 ± 0.048	0.241 ± 0.021	0.240 ± 0.015	0.278 ± 0.027	0.263 ± 0.054	0.215 ± 0.020
Ka	0.035 ± 0.003	0.028 ± 0.004	**0.088 ± 0.015**	0.030 ± 0.005	0.034 ± 0.003	0.039 ± 0.005	**0.062 ± 0.014**	0.027 ± 0.004
Ka/Ks	0.150 ± 0.017^†^	0.125 ± 0.024	**0.374 ± 0.100**	0.125 ± 0.022	0.142 ± 0.016^†^	0.139 ± 0.023	**0.234 ± 0.072**	0.127 ± 0.024

* Out-of-frame sequences were excluded.

Mol., molecular

No., number

nt, nucleotides

Ks, Synonymous substitutions

Ka, Non-synonymous substitutions

^† ^P_Z-Test _<0.001 for purifying selection hypothesis (Ka/Ks <1).

^&^Value ± Standard Error.

Bold print highlights the higher molecular distance, Ka and Ka/Ks observed for segment 2, compared to the entire gene and to segments 1 and 3.

**Figure 3:**
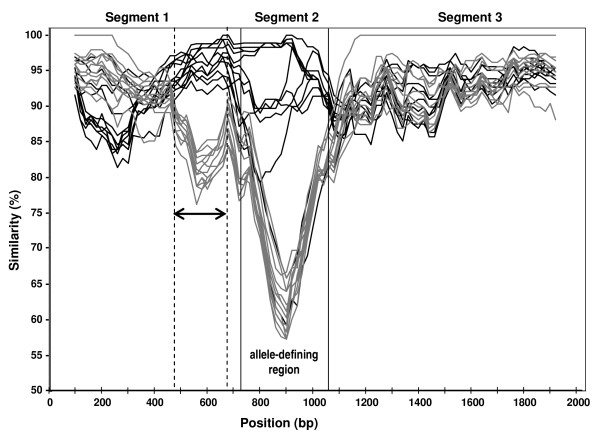
**Similarity plot representation of *homB *(black lines) and *homA *(grey lines) genes of various *Helicobacter pylori *strains**. The plot was generated by using 16 strains representative of each gene, with the Jukes-Cantor correction (1-parameter), a 200-bp window, a 20-bp step, without Gap Strip and the *jhp870 *gene sequence as reference (GenBank accession number NC_000921). The arrow delineates the region which discriminates between *homB *and *homA *genotypes. bp, base pair.

Analysis of the similarity plot of the 124 nucleotide sequences of *homB *and *homA *genes showed the existence of three distinct regions in both genes, named segments 1, 2 and 3, corresponding to the 5, middle and 3' regions of the genes, respectively (Fig. 3). The analysis performed independently on the three segments of each gene showed that segment 2 displayed the highest molecular distance as well as the highest Ka, even when compared to the entire gene (Table 1). These results were confirmed by the analysis of the nucleotide substitution rate over a sliding window, which also showed a significant increase in the Ka in segment 2 of *homB *gene. In fact, the mean Ka for this region (0.191 ± 0.059) was five fold higher than for the rest of the gene (0.037 ± 0.023). The same result was observed for *homA *gene (data not shown). These observations reveal a higher level of diversity of segment 2 in both genes.

**Figure 4:**
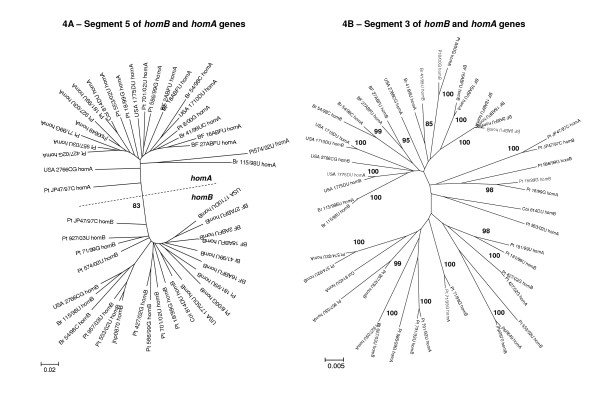
**Phylogenetic analysis of (A) segment 1 (nucleotides 1 to 750) and (B) segment 3 (nucleotides 1000 to 2000) for the pairs of *homB *and *homA *genes of 24 *Helicobacter pylori *strains carrying one copy of each gene**. The branch length index is represented below each tree. Country of origin is located at the beginning of each strain designation (Pt, Portugal; Br, Brazil; Col, Colombia; BF, Burkina Faso) followed by the *homB *or *homA *status. In Fig. 4A, the dotted line separates the *homB *and *homA *clusters. The numbers next to the main nodes are bootstrap values over 75% after 1000 iterations.

A phylogenetic analysis on each gene segment of 24 strains carrying one copy of each gene was also performed. The phylogenetic reconstruction of segment 1 showed that *homB *presented the highest similarity between orthologous genes, i.e., each *homB *was closely related to the *homB *in the other strains (Fig. 4A). A similar result was obtained for *homA *gene (Fig. 4A). In contrast, for segment 3, each *homB *was strongly correlated with the corresponding *homA *present in the same strain, indicating similarity between paralogous genes (Fig. 4B). The mean molecular distance and mean synonymous and non-synonymous substitution rates were calculated for all possible pairs of paralogous and orthologous genes, within the same strain and between strains. As expected, for segment 1, molecular distance and mean substitution rates were similar for pairs of *homB *and *homA *sequences in general. In contrast, for segment 3, these parameters were significantly lower between *homB *and *homA *sequences within the same strain than among different strains (Table 2). Additionally, for segment 3, molecular distance and nucleotide substitution rates were similar within each gene and between genes, indicating a parallel evolution of this segment in both genes, while for segment 1 those parameters were higher between genes than within each gene, pointing to an independent and divergent evolution of this segment in each gene (Table 3). Analysis of segment 2 was not conclusive, since clustering of *homB *and *homA *sequences was related to the allelic variant of the gene (see below).

**Table 2:** Analysis of molecular distances and synonymous and non-synonymous nucleotide substitutions in gene segments 1 and 3, between *homB *and *homA *(*homB *vs *homA*), within the same strain (intrastrain) and within different strains (interstrain), considering pairs of *homB *and *homA *sequences of 24 *Helicobacter pylori *strains.

	*homB *vs *homA*			
	
	Segment 1 (n = 48)		Segment 3 (n = 48)	
		
	**Intrastrain **^**a**^	**Interstrain **^**b**^	**Intrastrain **^**a**^	**Interstrain **^**b**^
Mol. distance (nt)	0.100 ± 0.012^&^	0.113 ± 0.010	0.020 ± 0.004	0.064 ± 0.004 ^c^
Ks	0.241 ± 0.048	0.286 ± 0.034	0.051 ± 0.013	0.202 ± 0.019 ^d^
Ka	0.061 ± 0.012	0.067 ± 0.011	0.010 ± 0.004	0.026 ± 0.004 ^e^
Ka/Ks	0.254 ± 0.071	0.234 ± 0.047	0.202 ± 0.093	0.130 ± 0.023

Mol., molecular

nt, nucleotides

Ks, Synonymous substitutions

Ka, Non-synonymous substitutions

^&^Value ± Standard Error.

^a ^All 48 sequences, totalling 24 comparisons.

^b ^All 48 sequences, totalling 552 comparisons (each *homB *was compared to each *homA*, excluding the pairs within the same strain)

^c ^Student's *t-test*, *p *< 10^-14 ^for interstrain vs intrastrain comparisons of molecular distance for *homB *and *homA *segment 3.

^d ^Student's *t-test*, *p *< 10^-10 ^for interstrain vs intrastrain comparisons of Ks for *homB *and *homA *segment 3.

^e ^Student's *t-test*, *p *< 10^-3 ^for interstrain vs intrastrain comparisons of Ka for *homB *and *homA *segment 3.

**Table 3:** Analysis of molecular distances and synonymous and non-synonymous nucleotide substitutions in gene segments 1 and 3, within each gene (*homB *or *homA *alone) and between genes in different strains (*homB *vs *homA*), considering pairs of *homB *and *homA *sequences of 24 *Helicobacter pylori *strains.

	Segment 1 (n = 24)			Segment 3 (n = 24)		
		
	***homB *alone **^**a**^	***homA *alone **^**a**^	***homB *vs *homA ***^**b**^	***homB *alone **^**a**^	***homA *alone **^**a**^	***homB *vs *homA ***^**b**^
Mol. distance (nt)	0.061 ± 0.006^&^	0.077 ± 0.007	0.113 ± 0.010	0.066 ± 0.005	0.065 ± 0.005	0.064 ± 0.004
Ks	0.199 ± 0.025	0.244 ± 0.026	0.286 ± 0.034	0.209 ± 0.020	0.207 ± 0.020	0.202 ± 0.019
Ka	0.026 ± 0.005	0.030 ± 0.004	0.067 ± 0.011	0.027 ± 0.005	0.025 ± 0.004	0.026 ± 0.004
Ka/Ks	0.131 ± 0.029	0.122 ± 0.021	0.234 ± 0.047	0.129 ± 0.027	0.121 ± 0.021	0.130 ± 0.023

Mol., molecular

nt, nucleotides

Ks, Synonymous substitutions

Ka, Non-synonymous substitutions

^&^Value ± Standard Error.

^a ^The 24 sequences, totalling 276 comparisons.

^b ^All 48 sequences, totalling 552 comparisons (each *homB *was compared to each *homA*, excluding the pairs within the same strain)

### Allelic variation

In both gene segments 1 and 3, the sequences were conserved between and within *homB *and *homA *genes (% of similarity >76% in segment 1 and >85% in segment 3) (Fig. 3). However, within segment 1, a region spanning from approximately 470 to 690 bp allowed the discrimination of *homB *and *homA *genes (arrow in Fig. 3). Gene segment 2, spanning from approximately 750 to 1050 bp in *homB *and from 720 to 980 bp in *homA*, was extremely polymorphic in both genes, with nucleotide differences being detected among the two genes and within sequences of the same gene from different strains (Fig. 3). This polymorphism is consistent with the highest nucleotide substitution rate observed for this gene segment.

**Figure 5:**
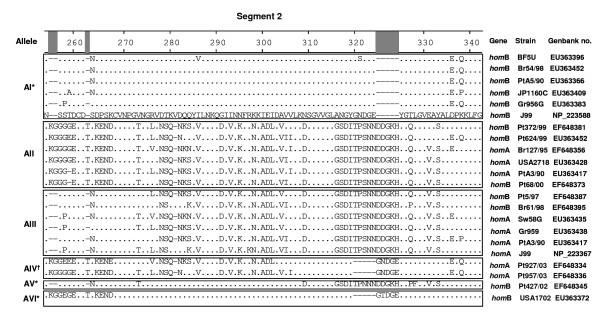
**Amino acid alignment of 22 *homB *and *homA *allelic region fragments from segment 2 (720 to 1050 bp; predicted amino acids 240 to 350), showing the six allelic variants**. The sequence of the *homB *product of the J99 strain was used as reference (Genbank accession number NP_223588). The dots refer to sites where the amino acids match those of the reference sequence, the hyphens represent deletions. The boxes are used to separate the 6 different allele groups named AI to AVI. Country of origin is located at the beginning of each strain designation (Pt, Portugal; Sw, Sweden; Gr, Germany; USA; Br, Brazil; Jp, Japan; BF, Burkina Faso). * Allelic variants exclusive of *homB*; ^† ^allelic variant exclusive of *homA*.

The detailed analysis of the previously mentioned 124 nucleotide and predicted amino acid sequences of segment 2 of *homB *and *homA *genes revealed the existence of six distinct and well conserved allelic variants, named AI, AII, AIII, AIV, AV and AVI (Fig. 5). The *homB *gene exhibited greater allelic diversity than *homA *gene, with five and three allelic variants, respectively. Two predominant allelic variants were observed: allele AI, detected in 78.9% of the *homB *sequences and exclusive of this gene, and AII, observed in 84.9% of *homA *sequences and in 11.3% of *homB *sequences. The four other allelic variants were less frequent: AIII was present in 4.2% and 11.3% of *homB *and *homA *genes, respectively; AIV was exclusively present in 3.8% of *homA *genes; and finally AV and AVI were exclusively present in 1.4% and 4.2% of *homB*, respectively.

Similarity plot analysis of *homB *and *homA *allelic sequences showed that the two predominant allelic variants of each gene, AI and AII, were the most distant groups (data not shown).

Interestingly, the closest variants to the *homB *predominant allele AI were the rarest variants AV and AVI, all three exclusive of *homB *gene. The closest variants to the *homA *predominant allele AII were AIII and AIV (data not shown).

Concerning the most prevalent *homB *and *homA *allele types, no geographical predominance of any allele was observed, and no correlation was found between any allelic variant and gastric disease as well (data not shown).

In order to test the *in vivo *expression of *homB *and *homA *allelic variants, human sera were tested with a recombinant purified HomB protein, allele type AI [9]. All sera (n = 24) showed an immunoreaction against this protein, suggesting that all *homB *and *homA *allelic variants are expressed during infection and are antigenic in humans. However, it should be noted that only one serum could be tested for the rarest allelic variants, AIII, AIV, AV and AVI.

## Discussion

In the present study, the distribution and diversity of two putative *H. pylori *OMP-coding genes, *homB *and *homA*, was evaluated in clinical strains with different geographical origins. Both genes displayed a varied worldwide distribution, with a marked difference between East Asian and Western countries, in accordance with other studies reporting such differences in the frequency of *H. pylori *virulence factors [16-19].

At least one copy of either *homB *or *homA *genes was found to be present in the genome of the *H. pylori *strains suggesting that these OMP-coding genes are under selective pressure to be maintained in the bacterium, as was reported for other *H. pylori *OMP-coding genes such as *babA*/*babB*, *sabA *and *oipA *[5-7]. Analysis of *homB *and *homA *genes revealed diversity regarding the number of copies and their genomic localization, regardless of the clinical origin of the strain, but with geographical specificity. Both the *homB*/*homA *single-copy and the double-copy genotypes were observed in Western strains while the East Asian strains presented the single-copy genotype only, suggesting that, if gene duplication had occurred, it did not seem to be a random event.

Variation in copy number of OMP-encoding genes can help the bacterium adapting to a particular host, which is essential to promote a chronic infection [5,11,20]. The fact that *homB *and *homA *genes display a high level of similarity, especially at the 5'and 3' ends, suggests that intra or intergenomic recombination events can occur, leading to gene duplication, deletion or *homB*/*homA *conversion, as a response to environmental changes. The presence of an intergenic region at the empty locus with high identity with both *homB *and *homA *suggests that the gene was lost, leaving short remnant sequences which will enable the gene to be integrated again by genomic recombination, in response to environmental changes, as has been hypothesized for other *H. pylori *genes [21,22].

Analysis of the *homB *and *homA *sequences revealed a complete ORF in the majority of the *H. pylori *strains tested, truncated genes being detected in only 5.7% of the cases. Interestingly, in three of the four out-of-frame *homB *sequences, the frameshift mutations occurred in short homopolymeric tracts, suggesting that *homB *displays phase variation and may be regulated by slipped-strand mispairing mechanism, which was not the case for the out-of-frame *homA *sequences. Phase variability has been reported to be a consistent marker for genes involved in niche adaptation and immune evasion [23,24]. Several *H. pylori *genes belonging to different functional classes have been established as phase variable genes [25,26], among which are OMP-encoding genes involved in adherence, such as *sabA *[6], *hopZ *[27], *babB *[28] and *oipA *[29]. HomB was previously found to contribute to *H. pylori *adherence [9]. Thus, the *on*/*off *switch of these genes would provide the bacterial population with a dynamic adherence pattern, as was experimentally demonstrated for *bab *adherence genes [20,28]. Based on the two mechanisms proposed for regulation of *homB *and *homA *gene expression, i.e., phase variation and intra/intergenomic recombination events, it can be speculated that these genes are implicated in the adaptation of *H. pylori *to its human host as well. However, the fact that only 5.7% of the strains have truncated *homA*/*B *sequences at loci A and B does not mean that the gene is not expressed *in vivo*. Indeed, the phase variation mechanism may allow the *in vivo *expression. Furthermore, the existence of a third locus, as was reported for *babA*/*B *[30], cannot be excluded, although previous hybridization experiments never revealed an additional locus [8,9].

Phylogenetic reconstruction of *homB *and *homA *genes was influenced by the geographical origin of the strains, with East Asian and Western strains showing the greatest divergence. This same clustering was observed for the paralogous genes *babA *and *babB *[31]. Overall, *homB *and *homA *displayed identical molecular mean distance at both nucleotide and amino acid levels. Nucleotide substitution rates were also similar for both genes suggesting that they are both subjected to parallel functional constraints. The segmental phylogenetic analysis showed the highest level of diversity for segment 2 of both genes, the middle allele-defining region, in comparison with the more conserved segments 1 and 3. This suggests that a higher degree of variation is allowed for segment 2, supporting the hypothesis that this gene segment is involved in the generation of antigenic diversity.

Another interesting point is that segment 3 of both *homB *and *homA *genes from the same strain clustered together in the phylogenetic tree, which is indicative of concerted evolution. This condition is observed when paralogous members of a gene family within a strain diverge at a slower rate than the homologous genes in other strains, and is a consequence of gene conversion events [32]. The evolutionary analysis of pairs of *homB *and *homA *sequences from the same strain also indicate that segment 3 of these genes is under concerted evolution, in contrast to segment 1 which displays a divergent evolution. Recently, Pride *et al*. showed that segment 3 of both *babA *and *babB *genes was under concerted evolution and demonstrated that the mechanism underlying this event was *babA*/*babB *conversion by intragenomic recombination [31]. Thus, the concerted evolution observed for segment 3 of *homB *and *hom*A genes supports the idea that they are involved in gene conversion events by intragenomic recombination. Since the rate of concerted evolution is expected to be higher when there are structural constraints [32], it is likely that segment 3 of *homA*/*homB *and *babA*/*babB *genes may encode portions of the protein that are essential for the function or for the structural integrity of those molecules.

Both *homB *and *homA *genes displayed allelic diversity in the middle region (segment 2), with *homB *exhibiting greater allelic diversity than *homA*. Allelic variation was also reported for other members of the *H. pylori *OMP family, such as *babA*/*babB *[33], *hopQ *[34] and *hopZ *[27] genes, which also share a conserved profile of gene segmentation, with the existence of at least two highly conserved allelic variants. In the case of *homB *and *homA *genes, no disease-associated allelic variant was observed nor was any allele associated with any particular virulence genotype or with the geographical origin of the strain. Instead, each gene presented a predominant worldwide allelic variant, present in up to 80% of the clinical strains, which may explain this lack of association. Moreover, it also suggests that the ability of the strain to adhere is not likely to be related to the allelic variant of the *homB *gene, as was demonstrated for the major *H. pylori *adhesin encoding gene *babA*. Indeed, it was reported that none of the five *babA *or the three *babB *allele groups is related to *cagA*, *vacA *or *iceA *genotypes or to the ability of the strain to bind to Lewis B antigen [33]. This would suggest that a greater allelic diversity may be more important in generating antigenic variation than in affecting the virulence of the strain. However, the detection of an immune reaction against a recombinant HomB protein of a single allelic variant, observed for all of the *homB *and *homA *allelic variants does not support this hypothesis. To clarify this issue, it would be interesting to evaluate the antigenicity against the six different HomB and HomA expressed alleles, especially using recombinant peptides containing only the allelic region (segment 2) of the gene, in order to exclude the presence of possible common epitopes outside the allelic determining region. Nevertheless, the results demonstrate that all allelic variants are expressed *in vivo*, which may contribute to the generation of new alleles through genomic recombination, increasing the fitness of the strains during human infection. A recombination event involving the duplicate genes encoding for the OMPs HopM and HopN, during human infection, which generated new alleles of these OMPs [21] is added proof.

## Conclusion

The results obtained in the present study suggest that *homB *and *homA *genes may be among the *H. pylori *OMP coding genes contributing to the mechanisms of *H. pylori *persistence, and would therefore be implicated in the development of disease.

## Methods

### Bacterial strains

A total of 455 *H. pylori *strains isolated from patients with upper gastrointestinal symptoms, from 10 different countries were included in the analysis. Table 4 summarizes the characteristics of the study population. Three *H. pylori *reference strains were used: 26695 strain (ATCC 700392), carrying one copy of *homA *gene (*HP0710*); HPAG1 strain, carrying one copy of *homB *gene (*HPAG1_0695*) and J99 strain (ATCC 700824), carrying one copy of each gene, *homA *(*jhp0649*) and *homB *(*jhp0870*) [12-14].

**Table 4:** Distribution of *Helicobacter pylori *strains included in the study (n = 455), according to the geographical origin, gender and patient's age.

Origin	No. of strains	Gender, % male	Median age ± SD (years)
**Western countries**			

Portugal	115	47.3	51.8 ± 15.4
France	35	82.9	47.7 ± 14.1
Sweden	27	58.8	66.6 ± 11.2
Germany	20	50.0	58.6 ± 11.9
USA	29	67.9	48.7 ± 12.0
Brazil	56	52.4	52.8 ± 16.4
Colombia	19	57.9	50.0 ± 12.7

**East Asian countries**			

Japan	72	57.9	44.3 ± 12.7
South Korea	71	76.1	44.7 ± 9.9

**African country**			

Burkina Faso	11	N.A.	N.A.

No., number

SD, standard deviation

N.A. not available

*H. pylori *strains were cultured from gastric biopsies on agar supplemented with 10% horse blood, preserved in Trypticase soy broth supplemented with 20% Glycerol and maintained at -80°C until used. Genomic DNA was extracted from a 48 h culture, using the QIAamp DNA mini kit (Qiagen GmbH, Hilden, Germany), according to the manufacturer's instructions.

### Genotyping of *homB *and *homA *by PCR and sequencing

A single PCR assay was used to discriminate between the *homB *and *homA *genes (fragments of 161 bp and 128 bp, respectively) [8]. In order to study the diversity of *homB *and *homA *genes, PCR primers targeting a conserved region of the flanking genes of both loci *jhp0649 *and *jhp0870*, according to the numbering of the J99 strain [13], were designed for amplification of the entire genes [8]. The fragments were subsequently sequenced using the PCR primers and internal primers, as previously described [8].

### Sequence analysis and phylogeny

Similarity plots, using SimPlot Version 3.5.1 http://sray.med.som.jhmi.edu/SCRoftware, were based on multiple alignments of the full nucleotide sequences of *homB *and *homA *genes generated by the BioEdit Sequence Alignment Editor (Version 7.0.1) [35]. Nucleotide sequences were translated using Translate Nucleic Acid Sequences software [36]http://biotools.umassmed.edu/cgi-bin/biobin/transeq. Neighbor-joining phylogenetic tree topologies of nucleotide and predicted amino acid alignments were constructed using the MEGA (Molecular Evolutionary Genetics Analysis) 3.1 software [37], on the basis of distances estimated using the Kimura two-parameter model [38]. This model corrects for multiple hits, taking into account transitional and transversional substitution rates. Branching significance was estimated using bootstrap confidence levels by randomly resampling the data 1000 times with the referred evolutionary distance model. Evolutionary parameters were determined using MEGA 3.1. Mean molecular distances were determined using the Kimura two-parameter method [38], while the overall mean of Ks and Ka substitutions were determined using the Nei-Gojobori method [39]. The standard error (SE) was determined for each parameter. A sliding window analysis of Ka and Ka/Ks ratio was performed using Swaap 1.0.2 software (Pride, D. T. (2000) Swaap - a tool for analyzing substitutions and similarity in multiple alignments). Due to the existence of alignment gaps, the complete-deletion option was used for all statistical analyses to normalize the number of differences on the basis of the number of valid sites compared. Bootstrap confidence levels were determined by randomly resampling the sequencing data 1000 times. The Codon Based Z-Test of selection [40] was used to evaluate the significance of the values for the ratio of non-synonymous to synonymous substitutions.

### In vivo expression of *homB *and *homA *allelic variants

A recombinant Glutathione S-transferase-HomB protein (rHpHomB), constructed with the complete *homB *allele type AI ORF, as previously described [9], was used to investigate the *in vivo *expression of the *homB *and *homA *allelic variants. Human sera, for which the corresponding strain was previously characterized with regard to *homB *or *homA *allelic variants, were used in Western-blot assays. Ten different human sera were tested for the two predominant *homB *and *homA *allelic variants AI and AII; only one serum was available for rarest allelic variants, AIII, AIV, AV and AVI, and was tested. All sera (n = 24) were obtained from adult patients (48.7 ± 6.9 years) presenting IgG antibodies against *H. pylori*, determined with the serological test Pyloriset EIA-G III (Orion Diagnostica, Espoo, Finland).

### GenBank accession numbers

The sequences used in this study are under the GenBank accession numbers [GenBanK: EF648331-EF648354, EU363366-EU363460 and EU910189-EU910194].

## List of Abreviations

(PUD): Peptic ulcer disease; (NUD): non-ulcer dyspepsia; (OMP): outer membrane protein; (ORF): open reading frame; (Ks): synonymous substitutions; (Ka): non-synonymous substitutions.

## Authors' contributions

MO carried out experimental design of the study, phylogenetic analysis and co-drafted the manuscript; RC carried out bacterial cultures, PCR and phylogenetic analysis; AM co-drafted the manuscript; YY and DQ carried out bacterial cultures and PCR; FM and LM supervised the study. All authors have read and approved the final version of the manuscript.
